# Relation between obesity-related comorbidities and kidney function estimation in children

**DOI:** 10.1007/s00467-022-05810-z

**Published:** 2022-11-22

**Authors:** Mark J. C. M. van Dam, Hans Pottel, Anita C. E. Vreugdenhil

**Affiliations:** 1 Centre for Overweight Adolescent and Children’s Healthcare (COACH), Department of Pediatrics, School of Nutrition and Translational Research in Metabolism (NUTRIM), Maastricht University Medical Centre +, Maastricht, The Netherlands; 2 Department of Public Health and Primary Care, KU Leuven Campus Kulak Kortrijk, Kortrijk, Belgium

**Keywords:** Childhood obesity, Creatinine, eGFR, Pediatrics, Obesity-related comorbidities

## Abstract

**Background:**

The current childhood obesity pandemic is likely to result in an increased risk of chronic kidney disease (CKD) later in life. Correlations between obesity-related comorbidities and kidney function can be found, but it is unclear to what extent this is caused by bias due to different mathematical forms of the estimated glomerular filtration rate (eGFR) equations. The present study aimed to analyze correlations between obesity-related comorbidities and different eGFR equations and to investigate whether rescaled serum creatinine (SCr/Q) for sex and age or height might be an alternative biomarker for kidney function estimation.

**Methods:**

This cross-sectional cohort study included 600 children with overweight and obesity. Mean age was 12.20 ± 3.28 years, 53.5% were female, and mean BMI *z*-score was 3.31 ± 0.75. All children underwent a comprehensive assessment that included anthropometrical and blood pressure measurements, laboratory examination, air displacement plethysmography, and polysomnography. Qage and Qheight polynomials were used to rescale SCr and multiple creatinine-based eGFR equations were compared.

**Results:**

SCr/Q and almost all GFR estimations significantly correlated with a waist-to-hip ratio, fat mass, homeostasis model assessment for insulin resistance, and triacylglyceride, HDL cholesterol, alanine transaminase, and serum uric acid concentrations. Multiple correlations, however, were not confirmed by all equations, which suggests dependency on the mathematical form of the different eGFR equations.

**Conclusions:**

Correlations between obesity-related comorbidities and creatinine-based eGFR are present in children with overweight and obesity, but depend to a large extent on the eGFR equation of choice. SCr/Q might be an alternative biomarker for assessing correlations between obesity-related comorbidities and kidney function in children with overweight and obesity.

**Graphical Abstract:**

**Figure:**
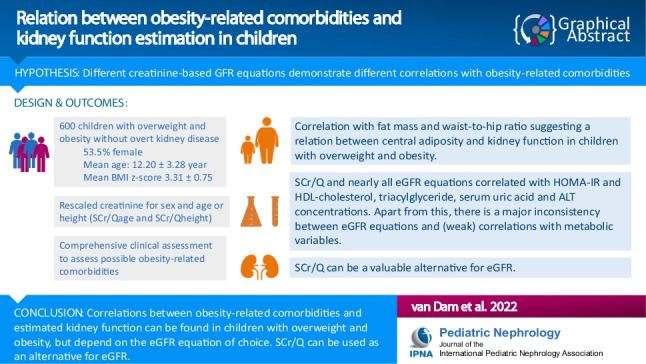
A higher resolution version of the Graphical abstract is available as Supplementary information

**Supplementary Information:**

The online version contains supplementary material available at 10.1007/s00467-022-05810-z.

## Introduction

The global prevalence of children with overweight and obesity has dramatically increased during the last few decades [1]. Obesity during childhood increases the risk of developing various comorbidities, including diabetes mellitus, hypertension, and dyslipidemia [2]. Additionally, children with obesity are prone to become adults with obesity, increasing the lifetime health risk even more [3]. With the first report that dates from 1974 describing an association between severe obesity and nephrotic-range proteinuria [4], nowadays, the relation between obesity and chronic kidney disease (CKD) is widely accepted [5–7]. Apart from the separate entity called obesity-related glomerulopathy, pathologically defined as glomerular hypertrophy and adaptive focal segmental glomerulosclerosis, obesity is an important prognostic factor for an adverse kidney outcome in patients with a solitary functioning kidney, IgA nephropathy, and autosomal dominant polycystic disease [8–10]. Obesity, therefore, plays a major role in the increasing prevalence of CKD, a condition that already afflicts 10% of the population worldwide [11].

The current pandemic of childhood obesity is likely to result in an increased risk of CKD in later life, as early-life adiposity is associated with CKD in the long term [12–14]. Unfortunately, kidney injury due to obesity might go unnoticed for years, hence named a “silent” comorbidity of obesity, with a consequence of delayed diagnosis and, therefore, impaired kidney prognosis.

Although the pathophysiology of obesity-related kidney injury is not fully understood, there seems to be a major role of glomerular hyperfiltration, increased activity of the renin-angiotensin-aldosterone system, insulin resistance, and lipid accumulation in the kidney [6, 7]. Since these alterations in the kidney occur before kidney dysfunction or the detection of microalbuminuria or hypertension [15], these markers might not be useful in the early phase of obesity-related kidney injury, and so there is need for other biomarkers. In children with obesity, urinary concentrations of neutrophil gelatinase-associated lipocalcin (NGAL) and kidney injury molecule-1 (KIM-1), both markers for proximal tubular cell injury, might be used for the early diagnosis of kidney injury [16]. In adolescents with obesity with a cystatin C-based glomerular filtration rate (GFR) ≤ 130 ml/min/1.73 m^2^, increased urinary NGAL concentrations might predict CKD [17]. Since these biomarkers are not (yet) implemented in clinical practice, most clinical studies on childhood obesity and kidney function use creatinine-based estimated glomerular filtration rate (eGFR) and (micro)albuminuria as markers for kidney health [13]. However, eGFR is almost always preserved in children with overweight and obesity without a history of kidney disease [18, 19], and microalbuminuria has a prevalence that ranges from 0.3 to 7.9%, which in fact is not different from the prevalence in lean children [20]. Clinical studies examining associations between obesity-related comorbidities and kidney function in childhood have yielded inconsistent results [13]. Important to consider is that eGFR equations using endogenous filtration markers are suffering from inaccuracy and imprecision [21], and not all creatinine-based GFR-estimating equations seem suitable for children with overweight and obesity [19]. Considering all of this, GFR-estimating equations might be inappropriate for evaluating correlations between kidney function and “metabolic health” in children with overweight and obesity. We postulate that the discrepant results in other studies examining associations between childhood obesity and kidney disease might be partially caused by bias and random error in eGFR equations. Therefore, in this study on children with overweight and obesity without overt kidney disease, we will compare different creatinine-based GFR-estimating equations and their correlations with anthropometric variables and obesity-related comorbidities. Additionally, we will examine whether rescaled SCr for sex and age or height might be used as an alternative marker.

## Methods

### Study population

This study uses baseline, pre-intervention data from the Centre of Overweight Adolescent and Children’s Healthcare (COACH): a clinical multidisciplinary lifestyle intervention program at the Maastricht University Medical Centre + (MUMC+). In this program, children with overweight, obesity, and severe obesity are evaluated to identify the underlying cause of the adiposity and potential obesity-related comorbidities, as described previously [19–22]. Between January 1, 2011, and April 1, 2019, 662 children entered the COACH program. Exclusion criteria for this study were age ≥ 18 years (*n* = 5), normal weight (at the moment of the measurements in our center (*n* = 5)), secondary cause of adiposity, known kidney disease (congenital or acquired), diabetes mellitus, and/or use of antihypertensive medication (*n* = 13). Serum creatinine (SCr) was not measured in 39 children. All other children (*n* = 600) were included in this study. The Medical Ethical Committee of the MUMC+ approved this study and informed consent was obtained.

### Clinical assessment, anthropometry, and body composition

After entering the COACH program, children were admitted for approximately 24 hours in the pediatric department of the MUMC+ for clinical evaluation. While children wore only underwear, weight and height were determined using a digital scale (Seca, Chino, CA) and stadiometer (De Grood Metaaltechniek, Nijmegen, the Netherlands), respectively. Weight and height were used to calculate body mass index (BMI) and BMI was converted into *z*-scores. Definitions from the International Obesity Task Force (IOTF) were used to define overweight, obesity, and severe obesity [23]. Waist and hip circumferences were determined, waist-to-hip ratio was calculated, and waist and hip circumference *z*-scores were obtained using reference values [24]. The equation described by Haycock et al. was used to calculate body surface area (BSA) [25]. In a subgroup of randomly chosen children (*n* = 285), fat mass and fat-free mass were determined by air displacement plethysmography (BodPod).

### Creatinine-based eGFR equations and microalbuminuria

Serum creatinine (SCr) was measured enzymatically (Cobas 8000, Roche). Since SCr is heavily dependent on sex, age, and height [26, 27], SCr was rescaled using Qage and Qheight polynomials obtained from the literature [28, 29]. SCr/Q (“rescaled SCr”) is sex and age or height independent for healthy subjects, depending on whether Qage or Qheight is used. In a previous study, it was concluded that this concept of rescaling SCr works very well in children with overweight and obesity [19]. Rescaled SCr was evaluated using the reference band [0.67–1.33], which represents the 2.5th and 97.5th percentiles [30]. The following SCr-based eGFR equations were evaluated, as described previously [19]: FAS-age [28]; FAS-height [29]; EKFC [31]; updated bedside Schwartz [32]; Schwartz-Lyon [33]; CKiDU25 [34]; LMR18 [35]; and CKD-EPI40 [36]. Urinary albumin was determined in a random urine spot and the urine albumin-to-creatinine ratio (UACR) was calculated. Microalbuminuria was present when the UACR was between 30 and 300 mg/mg.

### Laboratory analyses

Venous blood samples were collected while children were in a fasting state. Fasting plasma glucose, serum uric acid, total cholesterol, high-density lipoprotein (HDL) cholesterol, triacylglyceride, and alanine transaminase (ALT) concentrations were measured (Cobas 8000 modular analyzer, Roche). Serum insulin concentrations were analyzed (Immulite-1000, Siemens Healthcare Diagnostics). The Friedewald equation was used to calculate low-density lipoprotein (LDL) cholesterol concentrations [37]. Glycated hemoglobin (HbA1c) concentrations were measured (fully automated HPLC Variant II, Bio-Rad Laboratories). In order to estimate the presence of insulin resistance, the homeostasis model assessment for insulin resistance (HOMA-IR) was calculated from fasting glucose and insulin concentrations in which $\text{HOMA}-\text{IR}=\frac{\mathrm f\mathrm a\mathrm s\mathrm t\mathrm i\mathrm n\mathrm g\;\mathrm g\mathrm l\mathrm u\mathrm c\mathrm o\mathrm s\mathrm e\left(\frac{\mathrm{mmol}}{\mathrm l}\right)\times\mathrm f\mathrm a\mathrm s\mathrm t\mathrm i\mathrm n\mathrm g\;\mathrm i\mathrm n\mathrm s\mathrm u\mathrm l\mathrm i\mathrm n(\frac{\mathrm{mU}}{\mathrm l})}{22.5}$ [38].

### Blood pressure

Blood pressure (BP) was measured during daytime, every 3 min for about 20 times, depending on the tolerability of the child (Mobil-O-Graph, I.E.M. GmbH, Stolberg, Germany). Mean systolic BP (SBP) and diastolic (DBP) were calculated and converted into percentiles using normative pediatric BP tables [39].

### Polysomnography

All participants received a full polysomnography, and definitions of apnea and hypopnea were used as described in the appropriate manual [40]. The apnea-hypopnea index (AHI) was calculated out of the average number of apneas and hypopneas per hour of total sleep time. The oxygen desaturation index (ODI) was calculated as the total of desaturations ≥ 3% per hour.

### Statistical analysis

Normally distributed data are presented as mean ± standard deviation, median (interquartile range) otherwise. In the case of normally distributed data, Pearson’s correlation coefficient was used, Spearman’s correlation coefficient otherwise. A *P*-value below 0.05 was considered statistically significant, and all *P*-values are two-tailed. No correction was performed for multiple testing and *P*-values should be considered accordingly. The high sample size (*n* = 600) in this study may be responsible for turning clinically insignificant correlations into statistically significant correlations. As a rule of thumb, we here consider correlations < 0.200 as negligible from a clinical point of view. Statistical analysis was performed using SAS 9.4 (SAS Institute Inc., Cary, NC, USA). Clinical Trial registration: (ClinicalTrial.gov; Registration Number: NCT02091544).

## Results

### Characteristics

In Table 1, anthropometrical and metabolic characteristics of the 600 included children are presented, stratified according to sex. Mean age was 12.20 ± 3.28 years (ranged from 2.61 to 17.88 years) and there were slightly more females compared to males. Mean BMI *z*-score was 3.31 ± 0.75 and 21.3% of the children were in the overweight category, and 44.7% and 34.0% in the obese and severe obese categories, respectively. In both sexes, mean BSA was about 1.78 m^2^, and the body composed of approximately 44% fat mass. Compared to females, males had a significant higher BMI *z*-score, hip circumference *z*-score, waist-to-hip ratio, fasting glucose and alanine transaminase (ALT) concentration, and apnea-hypopnea index (AHI) and oxygen desaturation index (ODI). A total of 94.7% and 96.5% of the children had SCr/Qheight and SCr/Qage within the [0.67–1.33] reference interval, respectively. On the other hand, the concentration of fasting insulin was significantly higher in females, and for triacylglyceride and LDL cholesterol concentration, there was a trend towards higher values in females compared to males.

**Table 1:** Clinical, anthropometrical, and metabolic characteristics of the children

Variable	Total (*n* = 600)	Females (*n* = 321)	Males (*n* = 279)	*P*-value
Age (years)	12.20 ± 3.28	12.45 ± 3.42	11.91 ± 3.10	0.044*
Sex (%)	-	53.5	46.5	-
BMI *z*-score	3.31 ± 0.75	3.13 ± 0.70	3.52 ± 0.77	< 0.001*
BSA (m^2^)	1.78 ± 0.43	1.77 ± 0.43	1.78 ± 0.43	0.849
Fat mass (%)^1^	44.07 ± 6.36	43.60 ± 6.36	44.55 ± 6.34	0.208
Waist circumference *z*-score	5.51 ± 2.28	5.39 ± 2.27	5.67 ± 2.28	0.150
Hip circumference *z*-score	4.04 ± 1.87	3.83 ± 1.79	4.28 ± 1.94	0.004*
Waist-to-hip ratio	0.92 ± 0.08	0.91 ± 0.08	0.95 ± 0.08	< 0.001*
SCr/Qage	0.99 ± 0.16	1.01 ± 0.15	0.97 ± 0.15	0.001*
SCr/Qage within [0.67–1.33]	96.5	96.0	97.1	0.508
SCr/Qheight	0.96 ± 0.17	0.98 ± 0.17	0.93 ± 0.15	0.001*
SCr/Qheight within [0.67–1.33]	94.7	93.8	95.7	0.294
Microalbuminuria (%)^2^	6.7	8.7	4.4	0.070
Average SBP percentile^3^	88 (73–96)	88 [71–96]	88 [73–95]	0.641
Average DBP percentile^3^	69 (50–84)	70 [51–84]	67 [49–83]	0.610
Fasting glucose (mmol/l)	4.31 ± 0.62	4.23 ± 0.64	4.40 ± 0.59	0.001*
Fasting insulin (mU/l)^4^	14.9 (9.4–22.7)	15.9 [10.7–23.8]	13.4 [8.2–20.8]	0.026*
HOMA-IR^4^	2.66 (1.72–4.23)	2.88 [1.83–4.29]	2.53 [1.48–4.19]	0.164
HbA1c (%)	5.26 ± 0.38	5.27 ± 0.39	5.25 ± 0.35	0.559
Total cholesterol (mmol/l)	4.27 ± 0.80	4.32 ± 0.79	4.21 ± 0.80	0.073
LDL cholesterol (mmol/l)	2.49 ± 0.71	2.55 ± 0.73	2.43 ± 0.67	0.050
HDL cholesterol (mmol/l)	1.26 ± 0.31	1.25 ± 0.29	1.28 ± 0.32	0.290
Triacylglyceride (mmol/l)	0.97 (0.71–1.34)	1.00 [0.74–1.37]	0.94 [0.66–1.30]	0.065
Uric acid (mg/dl)^5^	5.08 (4.41–5.93)	5.08 (4.41–5.93)	5.08 (4.41–6.36)	0.065
ALT (U/l)	22.0 (17.0–31.0)	20.5 [16.0–27.0]	25.0 [19.0–36.0]	0.001*
AHI^6^	1.9 (1.0–3.8)	1.6 [0.8–3.3]	2.4 [1.2–4.6]	0.001*
ODI^6^	2.5 (1.1–4.4)	2.0 [0.9–3.6]	3.2 [1.4–5.4]	0.001*

Data presented as mean ± standard deviation, median (interquartile range), and percentage (%)

^1^Data was available for 285 children (144 females, 141 males), percentage of total body weight. ^2^Data was available for 436 children (230 females, 206 males). ^3^Based on a median of 21 (20–23) measurements, data was available for 554 children (291 females, 263 males). ^4^Data was available for 416 children (226 females, 190 males). ^5^Data was available for 388 children (195 females, 193 males). ^6^Data was available for 450 children (245 females, 205 males). *P*-value is obtained with the two-sample *t*-test (continuous variables) or Fisher’s exact test (binary variables)

*BMI*, body mass index; *BSA*, body surface area; *SCr/Qage*, serum creatinine normalized using Qage polynomials; *SCr/Qheight*, serum creatinine normalized using Qheight polynomials; *SBP*, systolic blood pressure; *DBP*, diastolic blood pressure; *HOMA-IR*, homeostatic model assessment of insulin resistance; *HbA1c*, glycated hemoglobin; *LDL*, low-density lipoprotein; *HDL*, high-density lipoprotein; *ALT*, alanine transaminase; *AHI*, apnea-hypopnea index; *ODI*, oxygen desaturation index

As shown in Table 2, mean and median eGFR were 98.4–115.8 ml/min/1.73 m^2^ and 98.1–113.7 ml/min/1.73 m^2^ using the different creatinine-based GFR-estimating equations, respectively. Moreover, equations differed concerning 2.5th and 97.5th percentiles and range (minimum and maximum).

**Table 2:** Descriptive statistics of the performance of the different creatinine-based GFR-estimating equations

	Mean	SD	Median	Percentile 2.5	Percentile 97.5	Minimum	Maximum	Age/height based	Power coefficient
SCr/Qage	0.99	0.16	0.99	0.72	1.33	0.43	1.50	Age	*x*
SCr/Qheight	0.96	0.17	0.94	0.66	1.31	0.55	1.69	Height	*x*
eGFR (ml/min/1.73 m^2^)									
FAS-age	111.0	18.3	108.5	80.7	149.6	71.5	252.4	Age	− 1
FAS-height	115.8	20.7	113.7	82.2	162.6	63.4	196.8	Height	− 1
EKFC	103.9	11.5	107.7	76.5	120.1	67.3	139.2	Age	*x*
CKiD	113.7	20.1	111.4	79.1	159.1	66.1	189.1	Height	− 1
Schwartz-Lyon	103.4	18.0	101.5	72.1	141.7	58.9	186.0	Height	− 1
CKiDU25	106.5	18.2	104.2	76.7	144.8	64.2	226.6	Height	− 1
LMR18	98.4	11.4	98.1	75.3	120.2	67.0	144.4	Age	x
CKD-EPI40	100.4	13.8	103.1	69.9	120.9	60.9	146.7	Age	x

*SCr/Qage*, serum creatinine normalized using Qage polynomials; *SCr/Qheight*, serum creatinine normalized using Qheight polynomials; *eGFR*, estimated glomerular filtration rate; *FAS*, full-age spectrum; *EKFC*, European Kidney Function Consortium; *CKiD*, chronic kidney disease in children; *CKiDU25*, CKiD under 25 years; *LMR18*, revised Lund-Malmö extended to children; *CKD-EPI40*, Chronic Kidney Disease Epidemiology Collaboration extended to children

In Supplementary Information Tables S1 and S2, anthropometrical and metabolic variables are compared between children with SCr/Qage and SCr/Qheight within the [0.67–1.33] reference range and values below 0.67 and above 1.33. As shown, groups are small and no multiple testing correction was performed, so the significance of the differences between the groups should be interpreted with caution.

### Correlation analyses

As presented in Table 3, SCr is correlated with age, weight, height, body surface area (BSA), fat mass, waist and hip circumference *z*-score, and waist-to-hip ratio. In children between the ages of 2 and 14 years, however, SCr linearly increases with age with no differences between males and females. From the age of 14 years, SCr begins to differ between sexes and ends on a plateau value of 0.70 mg/dl for adult females and 0.90 mg/dl for adult males. Since it is clear that SCr correlates with age and sex, SCr was rescaled using Qage polynomials. Moreover, we rescaled SCr using Qheight polynomials as well. Correlations between SCr and age, weight, height, BSA, and hip circumference *z*-score disappear when SCr is rescaled using Qage. SCr/Qheight however is still correlated with weight, height, and BSA. Correlations between SCr/Q (both Qage and Qheight) and fat mass are becoming more pronounced, and there is still a correlation with waist circumference *z*-score and waist-to-hip ratio. SCr/Qheight was weakly inversely correlated with BMI *z*-score (*r* = − 0.109, *P* = 0.007). SCr and SCr/Qage did not correlate with BMI *z*-score. All eGFR equations correlated with fat mass and waist-to-hip ratio, whereas for the other anthropometric variables, there was no consistency in correlations with eGFR.

**Table 3:** Correlations between SCr, SCr/Q, and creatinine-based eGFR equations and clinical and anthropometric variables in children

	Age (years)	Weight (kg)	Height (cm)	BMI *z*-score	BSA (m^2^)	Fat mass (%)^1^	Waist circumference *z*-score	Hip circumference *z*-score	Waist-to-hip ratio
SCr (mg/dl)	0.732**	0.611**	0.688**	− 0.037	0.649**	− 0.139*	0.321**	0.445**	− 0.237**
SCr/Qage	− 0.034	− 0.027	− 0.001	− 0.037	− 0.023	− 0.284**	− 0.105*	0.017	− 0.238**
SCr/Qheight	0.100*	− 0.093*	− 0.126*	− 0.109*	− 0.098*	− 0.208**	− 0.148**	− 0.065	− 0.207**
eGFR (ml/min/1.73 m^2^)									
FAS-age	0.047	0.037	0.004	0.040	0.031	0.296**	0.114*	− 0.010	0.248**
FAS-height	− 0.061	0.143*	0.160**	0.123*	0.144*	0.228**	0.189**	0.099*	0.212**
EKFC	− 0.044	− 0.039	− 0.034	0.027	− 0.036	0.262**	0.053	− 0.072	0.258**
CKiD	− 0.431**	− 0.280**	− 0.302**	0.088*	− 0.295**	0.224**	− 0.083*	− 0.206**	0.240**
Schwartz-Lyon	− 0.333**	− 0.179**	− 0.189**	0.098*	− 0.189**	0.230**	− 0.003	− 0.117*	0.281**
CKiDU25	− 0.107*	0.016	0.009	0.166**	0.013	0.267**	0.132*	0.037	0.290**
LMR18	− 0.034	− 0.027	− 0.022	0.036	− 0.024	0.292**	0.062	− 0.061	0.280**
CKD-EPI40	− 0.050	− 0.041	− 0.031	0.038	− 0.037	0.272**	0.055	− 0.068	0.277**

Data presented as Pearson’s correlation coefficient for normally distributed data and Spearman’s correlation coefficient otherwise

^1^Presented as percentage of the total body weight, data was available for 285 children

^*^P-value < 0.05. **P-value < 0.001

*BMI*, body mass index; *BSA*, body surface area; *SCr*, serum creatinine; *SCr/Qage*, serum creatinine normalized using Qage polynomials; *SCr/Qheight*, serum creatinine normalized using Qheight polynomials; *eGFR*, estimated glomerular filtration rate; *FAS*, full-age spectrum; *EKFC*, European Kidney Function Consortium; *CKiD*, chronic kidney disease in children; *CKiDU25*, CKiD under 25 years; *LMR18*, revised Lund-Malmö extended to children; *CKD-EPI40*, Chronic Kidney Disease Epidemiology Collaboration extended to children

In Table 4, correlations are shown between SCr, SCr/Q, and different creatinine-based eGFR equations and metabolic parameters. SCr/Qage and nearly all eGFR equations correlated with HOMA-IR and HDL cholesterol, triacylglyceride, serum uric acid, and ALT concentrations. Results are similar for SCr/Qheight with a few exceptions. Apart from this, there is a major inconsistency between eGFR equations and (weak) correlations with metabolic variables.

**Table 4:** Correlations between SCr, SCr/Q, and creatinine-based GFR-estimating equations and metabolic variables in children

	UACR (mg/g)^1^	Average SBP percentile^2^	Average DBP percentile^2^	Fasting glucose (mmol/l)	Fasting insulin (mU/l)^3^	HOMA-IR^3^	HbA1c (%)	Total cholesterol (mmol/l)	LDL cholesterol (mmol/l)	HDL cholesterol (mmol/l)	Triacyl-glyceride (mmol/l)	Serum uric acid (mg/dl)^4^	ALT (U/l)	AHI^5^	ODI^5^
SCr (mg/dl)	− 0.054	− 0.200**	− 0.152**	0.001	0.112*	0.102*	− 0.037	− 0.032	− 0.017	− 0.137*	0.079	0.469**	0.039	0.011	− 0.098
SCr/Qage	− 0.071	− 0.061	− 0.082	− 0.021	− 0.135*	− 0.114*	− 0.076	− 0.034	− 0.034	0.102*	− 0.122*	0.130*	− 0.135*	− 0.043	− 0.064
SCr/Qheight	− 0.098*	− 0.050	0.000	− 0.076	− 0.131*	− 0.137*	− 0.090*	0.047	0.030	0.158**	− 0.131*	0.081	− 0.105*	0.019	0.013
eGFR (ml/min/1.73 m^2^)															
FAS-age	0.112*	0.070	0.091*	0.026	0.145*	0.127*	0.072	0.037	0.038	− 0.090*	0.112*	− 0.147*	0.154*	0.070	0.095*
FAS-height	0.159*	0.050	0.0001	0.093*	0.149*	0.159*	0.086*	− 0.047	− 0.029	− 0.165**	0.133*	− 0.038	0.128*	− 0.008	− 0.002
EKFC	0.039	0.063	0.064	0.020	0.097*	0.076	0.073	0.015	0.014	− 0.082*	0.094*	− 0.147*	0.129*	0.052	0.065
CKiD	0.105*	0.148*	0.103*	0.028	0.015	0.019	0.058	0.0004	0.009	− 0.023	0.026	− 0.310**	0.043	− 0.012	0.010
Schwartz-Lyon	0.101*	0.123*	0.099*	0.052	0.067	0.068	0.065	−0.016	− 0.004	− 0.060	0.047	− 0.194**	0.102*	0.029	0.050
CKiDU25	0.103*	0.074	0.057	0.075	0.121*	0.118*	0.065	− 0.014	− 0.005	− 0.096*	0.087*	− 0.106*	0.183**	0.059	0.075
LMR18	0.055	0.073	0.080	0.039	0.111*	0.094	0.071	0.016	0.014	− 0.080	0.098*	− 0.150**	0.151**	0.061	0.080
CKD-EPI40	0.039	0.066	0.068	0.031	0.103*	0.084	0.073	0.014	0.015	− 0.082*	0.094*	− 0.138*	0.144*	0.063	0.078

Data presented as Pearson’s correlation coefficient for normally distributed data and Spearman’s correlation coefficient otherwise

^1^Data was available for 436 children. ^2^Based on a median of 21 (20–23) measurements, data was available for 554 children. ^3^Data was available for 416 children. ^4^Data was available for 388 children. ^5^Data was available for 450 children

^*^P-value < 0.05. **P-value < 0.001

*UACR*, urine albumin-to-creatinine ratio; *SBP*, systolic blood pressure; *DBP*, diastolic blood pressure; *HOMA-IR*, homeostatic model assessment of insulin resistance; *HbA1c*, glycated hemoglobin; *LDL*, low-density lipoprotein; *HDL*, high-density lipoprotein; *ALT*, alanine transaminase; *AHI*, apnea-hypopnea index; *ODI*, oxygen desaturation index; *SCr*, serum creatinine; *SCr/Qage*, serum creatinine normalized using Qage polynomials; *SCr/Qheight*, serum creatinine normalized using Qheight polynomials; *eGFR*, estimated glomerular filtration rate; *FAS*, full-age spectrum; *EKFC*, European Kidney Function Consortium; *CKiD*, chronic kidney disease in children; *CKiDU25*, CKiD under 25 years; *LMR18*, revised Lund-Malmö extended to children; *CKD-EPI40*, Chronic Kidney Disease Epidemiology Collaboration extended to children

## Discussion

In this study, we evaluated correlations between serum creatinine (SCr), rescaled SCr (SCr/Q), and different creatinine-based eGFR-estimating equations with anthropometric and metabolic variables in 600 children with overweight and obesity without overt kidney disease. SCr/Q height correlated with BMI *z*-score, whereas SCr and SCr/Qage did not. We verified the correlation between SCr and fat mass (and thus fat-free (lean) mass) [41], and this positive correlation becomes even more pronounced after rescaling SCr using Qage and Qheight polynomials. Moreover, we showed that all examined creatinine-based eGFR equations were positively correlated with fat mass and waist-to-hip ratio. Rescaled SCr and nearly all eGFR equations correlated with HOMA-IR and HDL cholesterol, triacylglyceride, serum uric acid, and ALT concentrations. Apart from this, there is a major inconsistency between eGFR equations and (weak) correlations with other metabolic variables. Based on this study, it is clear that the choice of a creatinine-based eGFR equation has an enormous impact on possible correlations with anthropometric variables and metabolic risk factors.

Obesity and excessive visceral fat are well-known cardiovascular and metabolic risk factors. The cluster of metabolic, anthropometric, and hemodynamic abnormalities is collectively known as metabolic syndrome. In children, there are at least 40 definitions of metabolic syndrome [42], and these definitions include parameters like waist circumference, triacylglyceride, HDL cholesterol and fasting glucose concentrations, and systolic blood pressure. In this study, we evaluated these and other highly prevalent obesity-related comorbidities such as hypertension, altered glucose metabolism and insulin resistance, dyslipidemia, hyperuricemia, non-alcoholic fatty liver disease (NAFLD), and sleep-disordered breathing. SCr/Q and nearly all eGFR equations correlated with HOMA-IR and HDL cholesterol, triacylglyceride, serum uric acid, and ALT concentrations, all well-known markers for insulin resistance, dyslipidemia, hyperuricemia, and NAFLD, respectively. Our findings therefore support the relation between metabolic syndrome and the kidney in children with overweight and obesity [43].

This study also demonstrates that the choice of a creatinine-based eGFR equation has a tremendous impact on correlations with the examined variables. Important to consider is that not all creatinine-based GFR-estimating equations seem suitable in children with overweight and obesity [19]. Equations with SCr in the denominator (with “1” as a power coefficient) may result in extremely high predictions. This is an artefact of these equations and exactly the reason why the EKFC equation has been developed. In this study, a difference in correlations with age-based and height-based creatinine-based eGFR equations was observed. The CKiD, CKiDU25, and Schwartz-Lyon equations are the only equations that correlate significantly with age. Moreover, the 2.5th percentile is larger than 75 ml/min/1.73 m^2^ for nearly all equations (except for Schwartz-Lyon and CKD-EPI40) and has previously been considered as the lower limit for GFR in children and adolescents [30]. Finally, the EKFC, LMR18, and CKD-EPI40 equations show a much lower “spread” (standard deviation) than the other equations, mainly because these equations use different power coefficients for SCr in their equation. This also results in much lower 97.5th percentile (and maximum) values.

Most other studies that examine the association between childhood obesity and kidney function use GFR-estimating equations or presence of microalbuminuria as markers for kidney function and body mass index (BMI) as a measure for adiposity [13]. In a recent review [13], 15 such studies were described and these studies found a mix of negative, positive, and no association between eGFR and BMI. In 10 studies, a Schwartz equation (most commonly the bedside Schwartz equation) was used for GFR estimation, and in 9 of these studies, a Schwartz equation was the only equation used. The bedside Schwartz equation, which was developed and validated in growth-retarded children with CKD, shows a significant eGFR decline with age and major differences between adolescent males and females in children with a GFR within the normal range [44]. Because of this, these Schwartz equations should not be used to examine associations between obesity and/or obesity-related comorbidities and kidney function in children without CKD.

All studies included in the review by Jadresic et al. used BMI as a marker for adiposity [13]. While BMI is the most commonly used measure for obesity, it gives no information on body fat content or distribution. Human body mass can be divided into two main compartments: fat mass and lean (fat-free) mass. BMI does not distinguish lean mass from fat mass and it is known that the amount of adiposity correlates with cardiovascular risk factors independent from BMI [45]. Several methods are currently available for body composition assessment, and since field body composition methods (including anthropometrics, skinfolds, bio-electrical impedance analysis, and ultrasound) are still inferior to more sophisticated laboratory body composition methods [46], air displacement plethysmography was used in this study. Fat mass and waist-to-hip ratio, a surrogate marker for visceral fat, were correlated with SCr/Q and all examined creatinine-based GFR-estimating equations. These findings support the unhealthy aspect of visceral adiposity and suggest a relation between central adiposity and kidney function in children with overweight and obesity.

Due to the cross-sectional study design and lack of measured GFR, it is not possible to state which correlations are clinically relevant. However, we suggest that rescaling SCr (either using Qage or Qheight polynomials) can be a valuable add-on next to creatinine-based GFR-estimating equations. Advantages of SCr/Q compared to eGFR equations are:

- There is no influence of statistical modeling to convert SCr to an eGFR formula
- There is a clear target value (namely SCr/Q = 1) for a healthy child, independent from age and sex, and a clear reference interval of [0.67–1.33]
- SCr/Qage (and thus eGFR FAS-age) might be directly added to the serum creatinine result of a patient in the clinical laboratory report, as height is often unavailable in the clinical laboratory. In this manner, interpretation of serum creatinine becomes more convenient for paediatricians
- Repeated measurements of SCr/Q over time are (probably) not influenced by age, sex, and other factors (like body surface area (BSA))
- Because SCr/Q is independent of BSA, it may serve as an excellent kidney function marker for longitudinal follow-up of children with overweight or obesity during a lifestyle program
- SCr/Q correlates with fat mass, waist-to-hip ratio, serum uric acid, and ALT, variables that (almost) all examined eGFR equations are correlated with to more or less the same degree

The question whether childhood obesity is already related to or leads to future kidney disease can obviously not be answered in this cross-sectional study. While there are some examples of prospective studies that address these questions [12, 47–49], data on this are scarce. We suggest that future, prospective studies include SCr/Q as a kidney biomarker and compare our findings with measured GFR. Moreover, we suggest to include biomarkers for detection of early kidney damage due to obesity, such as urinary NGAL and KIM-1 [7].

In conclusion, correlations between obesity-related comorbidities and creatinine-based eGFR can be found in children with overweight and obesity, but depend to a large extent on the eGFR equation of choice. SCr/Q is independent of the equation of choice and might be an alternative biomarker for assessing correlations between obesity-related comorbidities and kidney function in children with overweight and obesity.

## Supplementary Information

Below is the link to the electronic supplementary material.

Graphical Abstract (PPTX 55 KB)

Supplementary file2 (DOCX 27 KB)
