# Supplementary material for: Relation between obesity-related comorbidities and kidney function estimation in children
Source: Pediatr Nephrol. 2022 Nov 22;38(6):1867–76. doi: 10.1007/s00467-022-05810-z (PMC10154263; doi:10.1007/s00467-022-05810-z)
Supplement: Supplementary file 1 — Graphical Abstract (PPTX 55 KB) [file 467_2022_5810_MOESM1_ESM.pptx]

## Slide 1
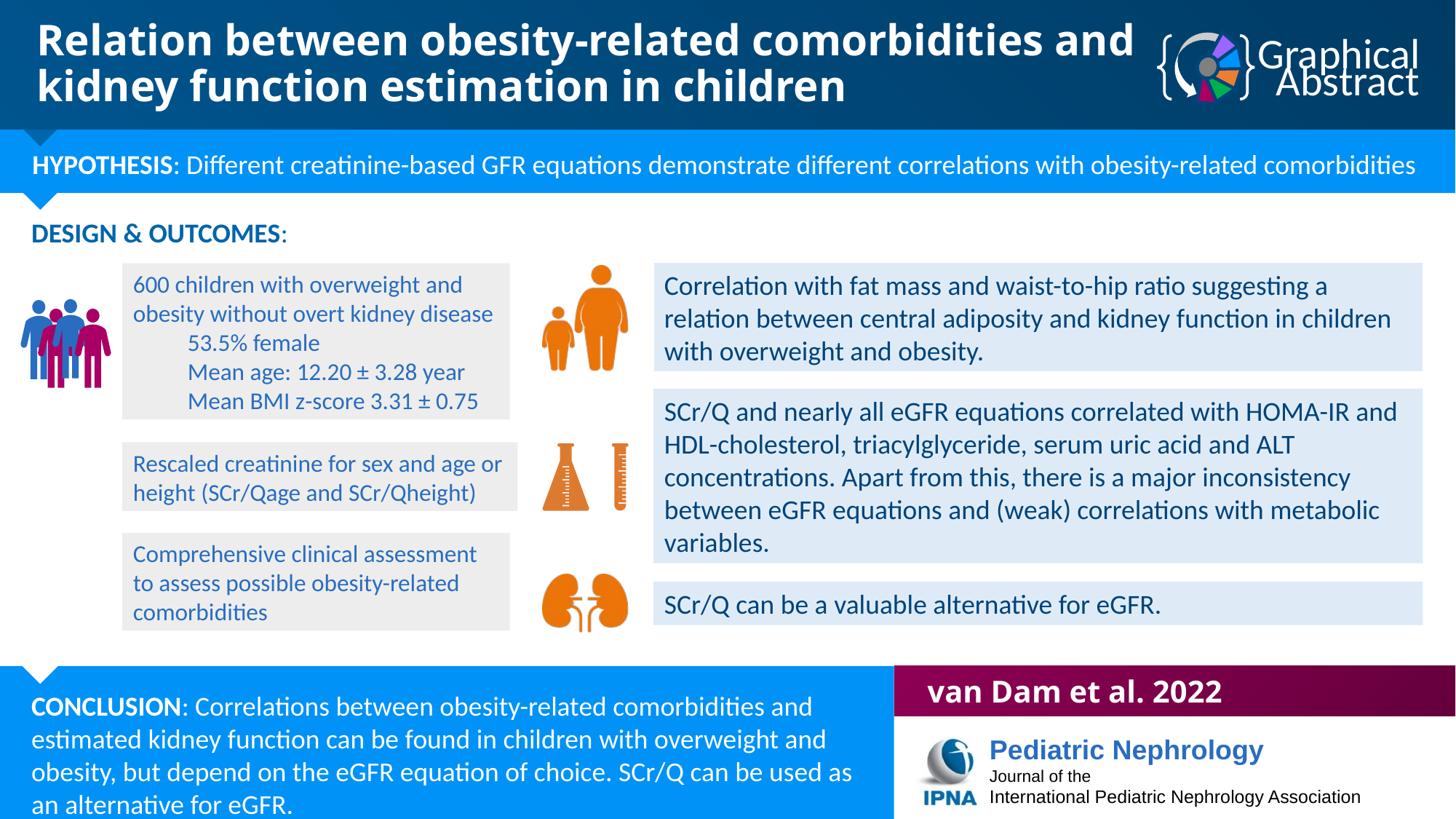

Relation between obesity-related comorbidities and
kidney function estimation in children
HYPOTHESIS: Different creatinine-based GFR equations demonstrate different correlations with obesity-related comorbidities
DESIGN & OUTCOMES:
Correlation with fat mass and waist-to-hip ratio suggesting a relation between central adiposity and kidney function in children with overweight and obesity.
600 children with overweight and obesity without overt kidney disease
53.5% female
Mean age: 12.20 ± 3.28 year
Mean BMI z-score 3.31 ± 0.75
SCr/Q and nearly all eGFR equations correlated with HOMA-IR and HDL-cholesterol, triacylglyceride, serum uric acid and ALT concentrations. Apart from this, there is a major inconsistency between eGFR equations and (weak) correlations with metabolic variables.
Rescaled creatinine for sex and age or height (SCr/Qage and SCr/Qheight)
Comprehensive clinical assessment to assess possible obesity-related comorbidities
SCr/Q can be a valuable alternative for eGFR.
van Dam et al. 2022
CONCLUSION: Correlations between obesity-related comorbidities and estimated kidney function can be found in children with overweight and obesity, but depend on the eGFR equation of choice. SCr/Q can be used as an alternative for eGFR.
