# Supplementary material for: Relation between obesity-related comorbidities and kidney function estimation in children
Source: Pediatr Nephrol. 2022 Nov 22;38(6):1867–76. doi: 10.1007/s00467-022-05810-z (PMC10154263; doi:10.1007/s00467-022-05810-z)
Supplement: Supplementary file 2 — Supplementary file2 (DOCX 27 KB) [file 467_2022_5810_MOESM2_ESM.docx]

**Supplementary Information**

**Relation between obesity-related comorbidities and kidney function estimation in children**

Mark J.C.M. van Dam (MD) ^1^, Hans Pottel (PhD) ^2^, Anita C.E. Vreugdenhil ^1^ (MD, PhD)

1. *Centre for Overweight Adolescent and Children’s Healthcare (COACH), Department of Pediatrics, School of Nutrition and Translational Research in Metabolism (NUTRIM), Maastricht University Medical Centre +, Maastricht, The Netherlands*
2. *Department of Public Health and Primary Care, KU Leuven Campus Kulak Kortrijk, Kortrijk, Belgium*

**Corresponding author**

Mark J.C.M. van Dam, Centre for Overweight Adolescent and Children’s Healthcare (COACH), Department of Pediatrics, Maastricht University Medical Centre +, P. Debyelaan 25, 6229 HX Maastricht, The Netherlands. E-mail: mark.van.dam@mumc.nl

*Comparison between children with SCr/Q within the [0.67 – 1.33] reference range, SCr/Q >1.33 and <0.67*

94.7% and 96.5% of the 600 included children had a SCr/Qheight and SCr/Qage within the [0.67 – 1.33] reference interval, respectively. So, in only a minority of the children, SCr/Q was not within this reference range. While SCr/Q >1.33 (corresponding an eGFR-FAS of 81 ml/min/1.73m2) is indeed elevated, it still is debated whether an eGFR <75 ml/min/1.73m2 (corresponding with a SCr/Q >1.43) should not be considered to be abnormal in children [1]. Only 3 of the 600 children had a SCr/Qage >1.43 (values of 1.44, 1.49 and 1.50). Five children had a SCr/Qheight >1.43 (values of 1.45, 1.48, 1.51, 1.55, 1.69).

In the following Supplementary Information Tables S1 and S2 anthropometrical and metabolic variables are compared between children with SCr/Qage and SCr/Qheight within the [0.67 – 1.33] reference range and values below 0.67 and above 1.33. As shown, groups are small and no multiple testing correction was performed, so the significance of the differences between the groups, should be interpreted with caution. Compared to children with a SCr/Q within the [0.67 – 1.33] reference range and children with a SCr/Q above 1.33, children with a SCr/Q below 0.67 have a higher fat mass percentage and so a lower lean body (and thus muscle) mass. Since a lower muscle mass is associated with a lower SCr, the low SCr/Q is likely to reflect low muscle mass in these children.

**REFERENCE**

1. Pottel H, Hoste L, Delanaye P (2015) Abnormal glomerular filtration rate in children, adolescents and young adults starts below 75 mL/min/1.73 m(2). Pediatr Nephrol 30:821-828

**Supplementary Information Table S1. Clinical, anthropometrical and metabolic characteristics of the children stratified according to SCr/Qage within the [0.67 – 1.33] reference range, >1.33 or <0.67 respectively**

| **Variable** | **SCr/Qage within the [0.67 – 1.33] reference range (n=579)** | **SCr/Qage > 1.33**  **(n=14)** | **SCr/Qage < 0.67**  **(n=7)** | **P-value** |
| --- | --- | --- | --- | --- |
| Age (years) | 12.2 [10.0 – 14.9] | 11.3 [7.7 – 14.1] | 14.1 [8.1 – 16.5] | 0.455 |
| Female sex (%) | 53.2 | 71.4 | 42.9 | 0.341 |
| BMI z-score | 3.24 [2.79 – 3.81] | 3.16 [2.54 – 3.57] | 3.23 [2.65 – 3.65] | 0.535 |
| BSA (m^2^) | 1.77 [1.47 – 2.07] | 1.73 [1.29 – 2.01] | 1.91 [1.28 – 2.13] | 0.522 |
| Fat mass (%) ^1^ | 44.3 [40.2 – 48.7] | 40.5 [33.1 – 41.7] | 60 [60 – 60] | 0.063 |
| Waist circumference z-score | 5.20 [3.90 – 7.00] | 4.05 [3.65 – 5.38] | 6.00 [3.88 – 7.75] | 0.120 |
| Hip circumference z-score | 3.90 [2.70 – 5.30] | 3.25 [2.68 – 4.78] | 4.25 [2.63 – 4.45] | 0.356 |
| Waist-to-hip ratio | 0.92 [0.87 – 0.98] | 0.88 [0.83 – 0.91] | 1.01 [0.87 – 1.06] | 0.018* |
| Microalbuminuria (%) ^2^ | 6.4 | 0.0 | 40.0 | 0.008* |
| Average SBP percentile ^3^ | 87 [72 – 96] | 91 [75 – 97] | 95 [80 – 99] | 0.231 |
| Average DBP percentile ^3^ | 68.5 [49.0 – 84.0] | 75.0 [59.5 – 78.5] | 80 [75 – 93] | 0.139 |
| Fasting glucose (mmol/L) | 4.30 [3.90 – 4.70] | 4.25 [4.00 – 4.53] | 4.60 [3.90 – 4.90] | 0.143 |
| Fasting insulin (mU/L) ^4^ | 14.8 [9.4 – 22.7] | 14.8 [7.4 – 20.0] | 30.6 [10.9 – 69.1] | 0.577 |
| HOMA-IR ^4^ | 2.65 [1.73 – 4.22] | 2.20 [1.38 – 4.04] | 7.47 [2.08 – 15.49] | 0.579 |
| HbA1c (%) | 5.25 [5.10 – 5.50] | 5.15 [4.98 – 5.53] | 5.15 [5.08 – 5.30] | 0.630 |
| Total cholesterol (mmol/L) | 4.20 [3.70 – 4.80] | 4.25 [3.60 – 4.80] | 4.20 [3.80 – 5.20] | 0.950 |
| LDL-cholesterol (mmol/L) | 2.40 [2.00 – 2.90] | 2.50 [2.20 – 3.13] | 2.30 [2.10 – 3.30] | 0.952 |
| HDL-cholesterol (mmol/L) | 1.20 [1.05 – 1.40] | 1.30 [0.98 – 1.63] | 1.50 [1.00 – 1.60] | 0.220 |
| Triacylglyceride (mmol/L) | 0.97 [0.71 – 1.34] | 0.95 [0.68 – 1.22] | 0.75 [0.48 – 1.37] | 0.940 |
| Uric acid (mg/dL) ^5^ | 5.08 (4.41 – 5.93) | 5.00 [4.58 – 5.85] | 5.42 [4.75 – 6.09] | 0.437 |
| ALT (U/L) | 22.0 [17.0 – 30.8] | 22.0 [16.5 – 28.5] | 46.0 [18.0 – 74.0] | 0.896 |
| AHI ^6^ | 1.9 [1.0 – 3.8] | 1.85 [0.73 – 4.05] | 7.25 [3.58 – 59.60] | 0.118 |
| ODI ^6^ | 2.5 [1.1 – 4.3] | 1.70 [0.35 – 4.75] | 9.45 [6.35 – 73.23] | 0.117 |

*Data presented as median (interquartile range) and percentage (%). Abbreviations: BMI, body mass index; BSA, body surface area; SCr/Qage, serum creatinine normalized using Q-age polynomials; SCr/Qheight, serum creatinine normalized using Q-height polynomials; SBP, systolic blood pressure; DBP, diastolic blood pressure; BP, blood pressure; HOMA-IR, homeostatic model assessment of insulin resistance; HbA1c, glycated hemoglobin; LDL, low density lipoprotein; HDL, high density lipoprotein; ALT, alanine transaminase; AHI, apnea-hypopnea index; ODI, oxygen desaturation index. ^1^ Data was available for 285 children (only 1 child in the SCr/Qage <0.67 group), percentage of total body weight. ^2^ Data was available for 436 children. ^3^ Based on a median of 21 (20-23) measurements, data was available for 554 children. ^4^ Data was available for 416 children. ^5^ Data was available for 388 children. ^6^ Data was available for 450 children. P-value is obtained with the Kruskal Wallis test or Fisher’s exact test (binary variables). No multiple testing correction was performed.*

**Supplementary Information Table S2. Clinical, anthropometrical and metabolic characteristics of the children stratified according to SCr/Qheight within the [0.67 – 1.33] reference range, > 1.33 or < 0.67 respectively**

| **Variable** | **SCr/Qheight within the [0.67 – 1.33] reference range (n=568)** | **SCr/Qheight**  **> 1.33**  **(n=13)** | **SCr/Qheight**  **< 0.67**  **(=19)** | **P-value** |
| --- | --- | --- | --- | --- |
| Age (years) | 12.1 [9.7 – 14.7] | 15.6 [12.8 – 16.8] | 14.3 [11.7 – 15.9] | 0.010* |
| Female sex (%) | 53.0 | 76.9 | 52.6 | 0.231 |
| BMI z-score | 3.24 [2.79 – 3.78] | 3.32 [2.56 – 4.25] | 3.24 [3.05 – 3.99] | 0.867 |
| BSA (m^2^) | 1.75 [1.45 – 2.06] | 1.82 [1.60 – 2.06] | 2.21 [2.07 – 2.61] | 0.009* |
| Fat mass (%) ^1^ | 43.9 [40.0 – 48.3] | 41.0 [38.6 – 41.7] | 48.5 [44.8 – 60.4] | <0.001* |
| Waist circumference z-score | 5.10 [3.88 – 6.90] | 4.30 [3.60 – 9.55] | 7.00 [5.70 – 9.60] | 0.009* |
| Hip circumference z-score | 3.80 [2.65 – 5.15] | 3.70 [2.85 – 5.65] | 5.00 [4.20 – 6.50] | 0.050 |
| Waist-to-hip ratio | 0.92 [0.87 – 0.98] | 0.86 [0.83 – 0.95] | 0.94 [0.87 – 1.06] | 0.165 |
| Microalbuminuria (%) ^2^ | 6.0 | 11.1 | 23.1 | 0.045* |
| Average SBP percentile ^3^ | 88 [73 – 96] | 77 [69 – 96] | 83.5 [71.5 – 95.3] | 0.371 |
| Average DBP percentile ^3^ | 69 [50 – 84] | 75 [56 – 89] | 59.0 [34.5 – 78.5] | 0.866 |
| Fasting glucose (mmol/L) | 4.30 [3.90 – 4.70] | 4.15 [3.93 – 4.45] | 4.30 [4.00 – 5.25] | 0.416 |
| Fasting insulin (mU/L) ^4^ | 14.7 [9.2 – 22.3] | 17.9 [10.0 – 27.3] | 20.5 [11.5 – 44.5] | 0.520 |
| HOMA-IR ^4^ | 2.65 [1.71 – 4.19] | 3.04 [1.84 – 5.21] | 5.45 [2.09 – 10.90] | 0.815 |
| HbA1c (%) | 5.20 [5.00 – 5.50] | 5.40 [5.10 – 5.50] | 5.20 [5.08 – 6.53] | 0.451 |
| Total cholesterol (mmol/L) | 4.20 [3.70 – 4.80] | 4.70 [4.05 – 5.20] | 4.30 [3.60 – 4.80] | 0.293 |
| LDL-cholesterol (mmol/L) | 2.40 [2.00 – 2.90] | 3.00 [2.40 – 3.20] | 2.30 [1.90 – 3.00] | 0.186 |
| HDL-cholesterol (mmol/L) | 1.20 [1.10 – 1.40] | 1.30 [1.05 – 1.50] | 1.10 [1.00 – 1.30] | 0.555 |
| Triacylglyceride (mmol/L) | 0.97 [0.71 – 1.34] | 1.02 [0.80 – 1.41] | 1.09 [0.87 – 1.37] | 0.501 |
| Uric acid (mg/dL) ^5^ | 5.08 (4.41 – 5.93) | 5.76 [4.58 – 8.31] | 5.42 [4.49 – 5.72] | 0.591 |
| ALT (U/L) | 22.0 [17.0 – 30.0] | 26.0 [20.5 – 33.0] | 27.0 [18.0 – 52.0] | 0.277 |
| AHI ^6^ | 1.90 [1.00 – 3.80] | 2.90 [0.70 – 11.28] | 1.90 [0.90 – 5.00] | 0.390 |
| ODI ^6^ | 2.40 [1.10 – 4.40] | 3.70 [0.68 – 10.90] | 3.05 [1.23 – 5.28] | 0.194 |

*Data presented as median (interquartile range) and percentage (%). Abbreviations: BMI, body mass index; BSA, body surface area; SCr/Qage, serum creatinine normalized using Q-age polynomials; SCr/Qheight, serum creatinine normalized using Q-height polynomials; SBP, systolic blood pressure; DBP, diastolic blood pressure; BP, blood pressure; HOMA-IR, homeostatic model assessment of insulin resistance; HbA1c, glycated hemoglobin; LDL, low density lipoprotein; HDL, high density lipoprotein; ALT, alanine transaminase; AHI, apnea-hypopnea index; ODI, oxygen desaturation index. ^1^ Data was available for 285 children, percentage of total body weight. ^2^ Data was available for 436 children. ^3^ Based on a median of 21 (20-23) measurements, data was available for 554 children. ^4^ Data was available for 416 children. ^5^ Data was available for 388 children. ^6^ Data was available for 450 children. P-value is obtained with the Kruskal Wallis test or Fisher’s exact test (binary variables). No multiple testing correction was performed.*
